# The Journey toward Voluntary Public Health Accreditation Readiness in Local Health Departments: Leadership and Followership Theories in Action

**DOI:** 10.3389/fpubh.2015.00043

**Published:** 2015-03-03

**Authors:** Angela L. Carman

**Affiliations:** ^1^Department of Health Management and Policy, College of Public Health, University of Kentucky, Lexington, KY, USA

**Keywords:** accreditation, leadership, followership, public health

## Abstract

Local health department directors’ intent on getting their organizations ready for accreditation must embrace the blurring of leader/follower lines and create an accreditation readiness team fueled not by traditional leader or follower roles but by teamship.

## Introduction

The Institute of Medicine’s 2002 report “The Future of the Public’s Health in the 21st Century” made several recommendations for improving and building capacity of local public health agencies, including increased training for public health leaders and the creation of an accreditation system (1). In 2007, following recommendations by the Exploring Accreditation Steering Committee, the Public Health Accreditation Board (PHAB) was incorporated (2).

The mission of PHAB is to promote and protect the health of communities by advancing the quality and performance of all public health departments in the United States (3, 4). In 2009, PHAB conducted a national beta test in which 30 state, local, and tribal health departments, of varying sizes, completed the accreditation process and provided feedback on both the process and the accreditation standards. The national public health accreditation program was launched in late 2011 after beta test adjustments were completed (2). Other organizations, such as hospitals and schools, have utilized accreditation systems for many years, and while information regarding impact is limited, evidence is encouraging regarding positive changes in service quality and outcomes (5). The national public health accreditation system is the first national accreditation effort to measure performance and ensure quality public health services in local communities. Since difficult economic times cause health departments to adjust services and staff, the field of public health strains under the increasing pressures of infectious and chronic diseases, emergency preparedness concerns, and the loss of essential services (6). The underlying premise of national public health accreditation, performance improvement, has become increasingly important.

As local health department leaders and staff become appropriately educated about the PHAB standards and measures for accreditation and the technical assistance tools provided by many of public health’s national partners (3), the journey toward public health accreditation begins in the local health department. As this journey progresses toward application for accreditation and site survey (7), local health departments must evaluate their work against accreditation standards and identify both strengths and challenges. Through the creation of a highly functioning team, the concepts of leadership and followership theories are seen in action and knowledge gained from these concepts in the form of teamship will contribute toward successful accreditation readiness.

## Leadership

In the book, *Leadership: Theory and Practice*, Northouse defines leadership as “a process whereby an individual influences a group of individuals to achieve a common goal” (8). Leaders of local health departments must draw upon this concept of “influence” as they introduce accreditation to board and staff members. Accreditation concepts include an emphasis on the 10 essential public health services, organization-wide quality improvement, and measurements of performance (9). Such concepts are new to many individuals associated with local health departments, and leaders will need to understand how to elicit interest by their staff members and growth in their understanding of the accreditation process (10) in a program that has only been recently launched (2, 3). While many factors within communities and local health departments influence performance of local health departments, leadership is of critical importance (11, 12). Knowledge of the impact of leadership has evolved from the trait-based theories to more modern, contingency theories that add a focus on the follower (13). In this paper, a sample of evolving theoretical approaches to understanding leadership will be used to view the leader’s potential impact on the local health department’s readiness for national public health accreditation.

## The Leader as an Individual

In the past, the study of leadership focused on the personality of leaders implying that only specific personality types could be effective leaders. The personality perspectives of leadership focused on those qualities that leaders possess which enable them to influence specific behaviors in others (8). Kouzes and Posner, in a cross-cultural study, identified honesty, a forward-looking perspective, competence, and an ability to inspire as leadership qualities, which stand the test of time (14). Quinn categorizes critical leadership traits as having the ability to focus on others, being results-oriented, and being open to external signals that suggest needed change (15). Recognition of the impact adherence to accreditation standards can have on a local health department may be examples of external signals of change.

Examples of the personality perspectives or trait theories of leadership can be found in those leaders who have already prepared staff members for accreditation. By honestly explaining what is known about the PHAB process and by presenting accreditation as a proactive means of improving the provision of public health services (3), these individuals are leading their organizations into the future. These leaders are seen addressing the concerns of the 2002 IOM report regarding the future of the public’s health as they use their forward-looking perspectives to learn from the roadmap that accreditation provides in planning, assessment, and community collaboration for organizational improvement (16).

## The Leader Considers the Follower

In addition to the traits that leaders possess, there are a variety of theories that explain what specific leaders do to influence a group (8). These theories result in considering the person being led—the follower. In her book, *Followership: How Followers Are Creating Change and Changing Leaders*, Kellerman states that followers are “subordinates who have less power, authority and influence than do their superiors and who therefore usually, but not invariably, fall into line”. She defines the concept of followership as “the response of those in subordinate positions (followers) to those in superior ones (leaders)” (17).

The theory of transformational leadership involves the process a leader goes through to bring about the transformation of their followers and their methods of work. Transformational leadership includes a leader’s use of emotion, formation of long-term goals, and an assessment of the needs of followers (8, 18). True transformational leaders have an exceptional level of influence (8) as they strive to create a personal connection with individuals particularly during times of great change (19). It is through this personal connection that transformational leaders discover the strengths in those they work with and find ways to capitalize on them (10).

Examples of transformational leadership can be found as local health department leaders embrace changes in the field brought about by increasing community problems, decreasing financial support, and the advent of voluntary public health accreditation. Specific examples of transformational leadership can be seen in health department leaders forging new partnerships created through the accreditation readiness process. Leaders of small health departments with minimal resources have banded together with neighboring county and district health departments, universities, and other non-profit entities to work toward community health assessments (20) required as pre-requisites to accreditation (7). Such partnerships break down silos that have existed in the public health system, capitalizing on the strengths of each partner, and transforming the way the business of public health occurs (12, 21).

Other leadership theories include the situational leadership theory, in which leaders first understand the level of competence and commitment of their followers and then match their leadership styles to that level. Variations are seen in the level (high or low) of directive (task) behaviors in which the leader gives direction, establishes goals, sets timelines, and defines roles. Variations are also seen in the level (high or low) of supportive (relationship) behaviors in which the leader is concerned with follower’s feelings and communication (22).

Examples of situational leadership may be seen throughout the journey of accreditation readiness. Early steps in the process will involve high levels of direction as leaders must give assignments to followers as to who will serve on specific accreditation teams and which projects, either accreditation, pre-requisites, or programmatic performance improvement initiatives will occur first. During this period, high levels of supportive behaviors will also be needed from leaders as followers will be working on projects, using unfamiliar terms, and working with other individuals for the first time. However, as the educational level (competence) and the understanding and acceptance (commitment) of followers toward the accreditation process increase, leaders will be able to change levels of directive and supportive behaviors (23).

## The Follower

Bennis writes that followers are more important now than ever before as problems are more complex and solutions can only come through collaborative problem solving and process implementation at all levels of the organization (24). Accreditation readiness is a complex process unfamiliar to many local health department employees. In order to successfully complete the accreditation readiness checklist and associated responsibilities, all members of the organization will need to be involved in standard interpretation, data collection, and performance improvement (7). They will need to increase their comfort level with performance improvement and continually strive to perform their jobs more efficiently and effectively.

## The Follower as an Individual

Similar to the early study of leaders, the study of followers is often dedicated to the traits of good followers. In *In Praise of Followers*, Kelley writes that followers can be both effective and ineffective. Effective followers are enthusiastic, intelligent, and self-reliant in their pursuit of organizational goals. They dedicate themselves to increasing their job competence in order to maximize the impact on the organization. Kelley describes an effective follower as courageous, honest, and credible (25). The effective follower can also be referred to as the “star follower” who does not follow a leader blindly, but constructively questions processes and procedures in order to improve the organization (26). The star follower is ideal for an accreditation readiness effort as questioning processes and procedures is at the heart of performance improvement. Star followers in the accreditation readiness process can be seen gaining expertise, leading initiatives, and building teams.

In contrast to the trait theories or personality perspectives of leadership, which tend to be positive in nature, Kelley also identifies other types of followers who are negatively contrasted to the star follower. Alienated followers often began as star followers but allow a negative experience to cause them to become angry and withdrawn. Rather than assisting with improvement of the organization, the alienated follower often tears down what leaders or other followers are trying to build (26). In an accreditation readiness scenario of a local health department, an alienated follower can be very detrimental to the process. While intelligent enough to be a contributor, the negative attitude of an alienated follower will cause friction between the leader and other followers. As the leader portrays the benefits of accreditation as a means of demonstrating the value of public health to the community (3), the alienated follower will undermine this message by dwelling on perceived increased staff workload and costs to the organization or the unknown impact of accreditation on health outcomes. The alienated follower also represents a wasted source of job knowledge that could have been used in any aspect of the accreditation process.

Additional types of followers are Sheep (passive followers) and Yes-People (conformist followers). Both of these types of followers are passive. While the Yes-People or conformist followers are more involved in the workplace than the Sheep or passive followers, neither group are individuals who think for themselves. The Yes-People are dependent on their leaders for direction and often tell them only what they want to hear not the crucial information that they need to know (26). Both of these types of followers are detrimental to the accreditation readiness process as they withhold useful expertise in favor of waiting on the leader to direct them. In addition, the alienated, passive and conformist followers test, but not necessarily erode, the ability of the leader to transform the organization.

## The Leader with the Follower

The “command and control” form of leadership identified leaders as in control and followers as silent and subservient (13). This outdated approach has given way to blurred lines between leaders and followers in which leaders are asked to support their followers, and followers are frequently called upon to use judgment and critical thinking skills (27). In today’s workplace, designated leaders often follow and designated followers lead. According to Kellerman, the reason roles between leaders and followers reverse at times, often in the same day or with the same project, centers on competence (17). When competence was listed as one of the traits of personality perspective leadership theory (14), consideration was not given to the fact that a follower might be more competent in a specific area than the designated leader. When this is the case, leaders must employ the elements of situational leadership and adjust their leadership approach based on the competence and commitment of the follower (8). Thus situational leadership theory supports a leader’s delegation or blurring of traditional leader/follower roles, in the case of a competent and committed follower (23).

Townsend and Gebhardt refer to the blurring of lines between leader and follower roles as a continuum. At the extreme left of the continuum is “Capital L Leadership,” which indicates the formal leadership duties of organizational direction setting and resource allocation. Moving right on the continuum leads to “small L leadership” in which people skills play key role in getting people to do specific tasks (27). Another step to the right, finds Active Followership, which corresponds to Kelley’s “star follower” (26) concept and indicates an engaged follower (27). At the extreme left of the continuum is the passive follower who functions much like Kelley’s sheep or passive follower (26) and is completely unengaged (27).

However, Townsend and Gebhardt do not stop with just another method of classifying leaders and followers. They discuss what happens in the middle of the continuum where leaders and followers meet and the term of “teamship” is introduced. With the concept of “teamship,” they reinforce Kellerman’s theory of the periodic reversal of leader and follower roles. Teamship indicates interaction between leaders and followers. The focus is on a shared goal with members of the team assuming leader and follower roles according to the expertise needed by the team at any given moment of a project’s evolution (27). The transition between designated leader to team follower and back again is so seamless in true teamship that observers find it hard to identify the designated leader at all (27).

As local health departments engage in the accreditation readiness journey, health department leaders quickly realize that the tasks and responsibilities of accreditation are far reaching throughout the organization. Often trained as clinicians (28), health department leaders attempting to follow the road to accreditation need the expertise of varied disciplines that make up the health department staff. Many local health departments form accreditation readiness teams either dividing the responsibilities along the 10 essential public health services or PHAB domains or progressing through all aspects of accreditation in tandem together using expertise from all sources (29). Such accreditation readiness teams are excellent examples of teamship in which title or position in the organization is not the relevant factor. Instead the relevant factor is the expertise brought by the individual to the group.

## Conclusion

To lead an organization to improve its quality is a complex endeavor. Leadership can be analyzed according to leadership theories, which address the traits of leaders, the leaders’ ability to transform the people and systems with whom they work, or by the leader’s adjustment to follower needs. Each of these attempts to identify high quality leadership evidenced in an organization is complicated by the organizations environment, financial condition, rate of change in the industry, and by the followers with whom the leader works. Followers can be analyzed according to effective or ineffective traits and the impact on the leader of differing levels of follower commitment. However, both leaders and followers can have the most powerful impact on an organization, specifically a local health department beginning the journey toward accreditation readiness, when teamship is employed. Teamship occurs when leaders and followers interact, egos are set aside, and expertise and the job at hand dictate the changing roles of the members of the team. Leaders, such as local health department directors, who are intent on getting their organizations ready for accreditation must embrace the blurring of leader/follower lines and create an accreditation readiness team fueled not by traditional leader or follower roles but by teamship. This expanded expertise together with a genuine desire for organizational improvement provides the necessary tools for improvement and for success in the journey toward voluntary public health accreditation readiness.

## Conflict of Interest Statement

The author declares that the research was conducted in the absence of any commercial or financial relationships that could be construed as a potential conflict of interest.
